# Supplementary material for: Seasonal dynamics and molecular phylogenetic studies on cercariae in Central Zone of Kashmir valley
Source: PLoS One. 2025 Jul 10;20(7):e0325160. doi: 10.1371/journal.pone.0325160 (PMC12244628; doi:10.1371/journal.pone.0325160)
Supplement: S1 Raw Image — (DOCX) [file pone.0325160.s001.docx]

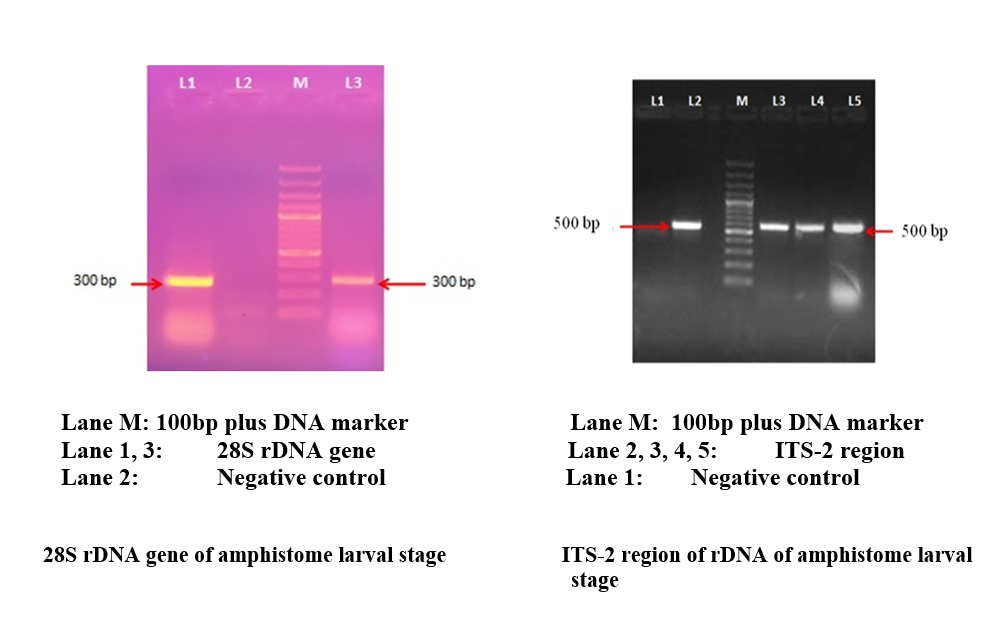

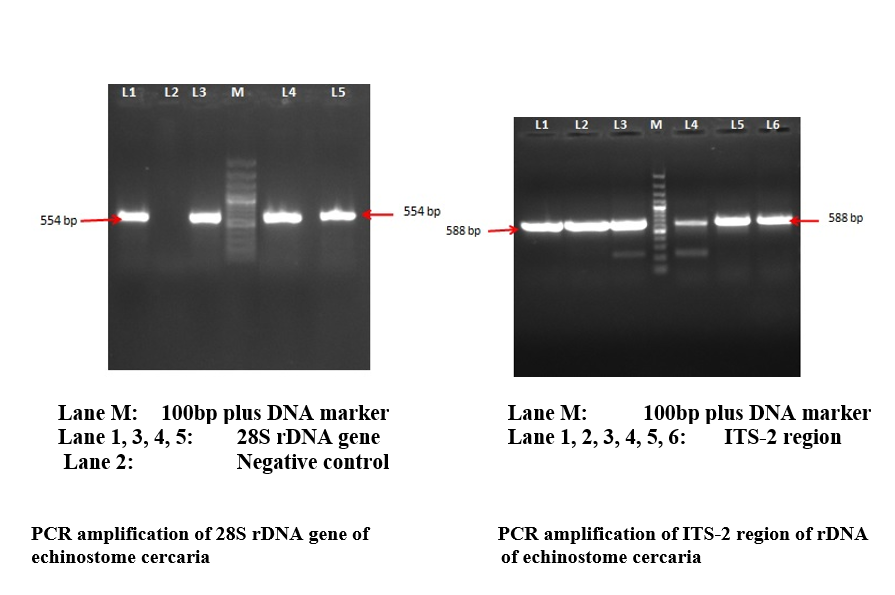


**ORIGINAL UNADJUSTED IMAGES OF GEL DATA ASKED AS “EDITS REQUESTED” UPON REVISED SUBMISSION**


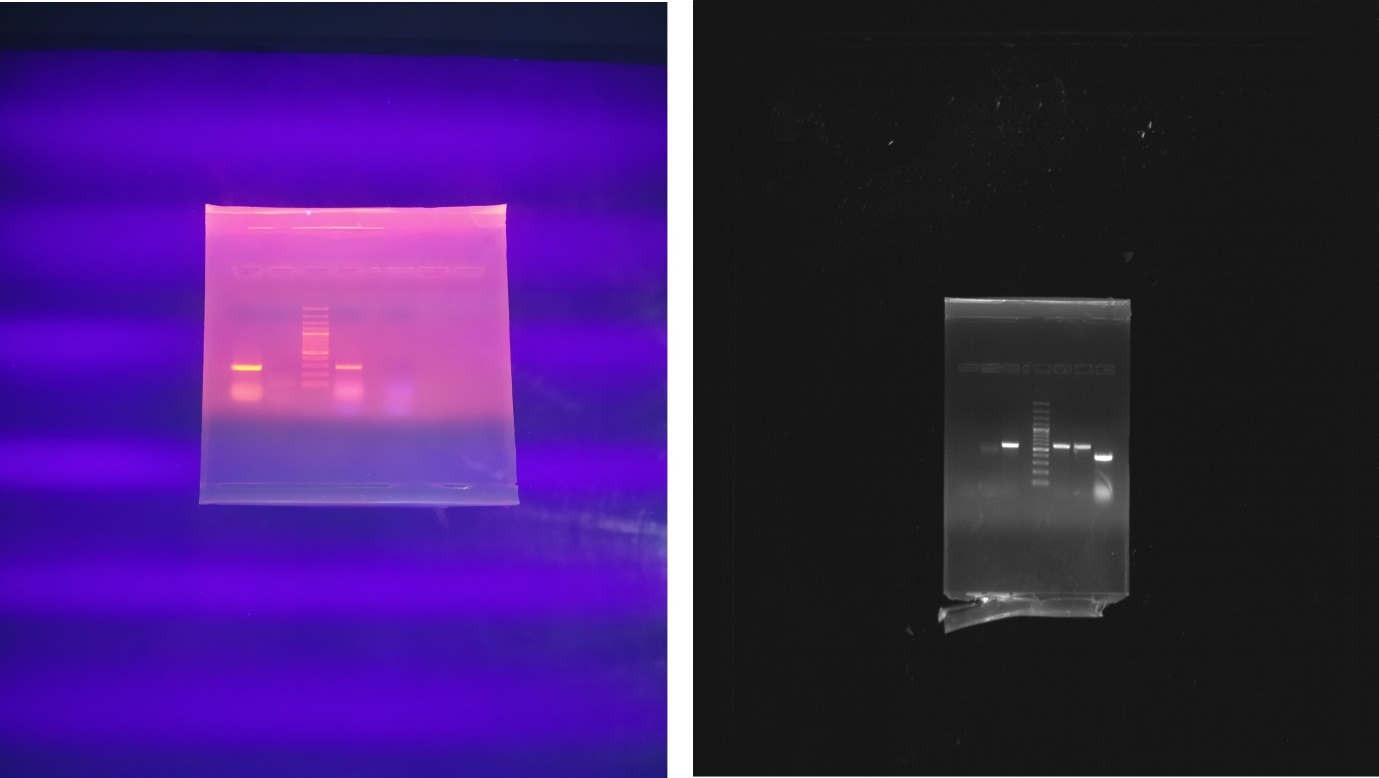


**28S rDNA gene of amphistome larval stage ITS-2 region of rDNA of amphistome larval stage**


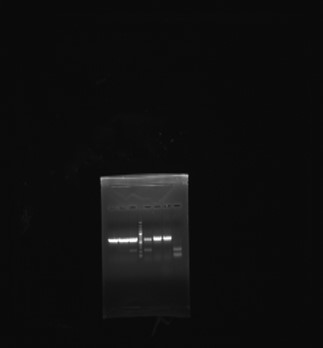


**PCR amplification of ITS-2 region of rDNA of echinostome cercaria**

**Uncropped original Gel image for PCR amplification of 28S rDNA gene of echinostome cercaria, has been misplaced unfortunately which is now unavailable. Sorry for inconvenience**
